# Estrogens from the Outside In: Alkylphenols, BPA Disrupt ERK Signaling *in Vitro*

**DOI:** 10.1289/ehp.119-a34b

**Published:** 2011-01

**Authors:** Julia R. Barrett

**Affiliations:** **Julia R. Barrett**, MS, ELS, a Madison, WI–based science writer and editor, has written for *EHP* since 1996. She is a member of the National Association of Science Writers and the Board of Editors in the Life Sciences

The body produces estrogens—including estrone (E_1_), estradiol (E_2_), and estriol (E_3_)—that direct reproductive system processes and contribute to the normal function of tissues including the brain, bone, and cardiovascular system. Certain xenoestrogens (estrogenic compounds introduced from outside the body) are suspected of disrupting these activities. In a new study, xenoestrogenic alkylphenols and bisphenol A (BPA) interfered with normal estrogenic signaling *in vitro*, which suggests they could disrupt normal physiologic function at critical life stages **[*EHP*** **119(1):104–112; Jeng and Watson]**.

Different estrogen receptors control different functions: receptors in the cell nucleus direct gene transcription, whereas receptors in the cell membrane direct signaling pathways via extracellular signal–regulated kinases (ERKs). ERK-controlled pathways respond to many biochemical stimuli and integrate these signals to direct a cell toward division, differentiation, death, or malignant transformation. The structurally related alkylphenols and BPA interact weakly with nuclear estrogen receptors, but they can have pronounced effects on signaling pathways mediated by estrogen receptors in the cell membrane.

In the current study, a rat pituitary cancer cell line was used to study the effect of alkylphenols and BPA on ERK1 and ERK2 activation (measured as phosphorylation), both alone and in combination with each physiologic estrogen. After treatment with each physiologic and environmental estrogen, the researchers measured time-dependent surges in ERK activation. In most cases, E_1_ and E_2_ prompted early, intermediate, and late surges in ERK activation at 5, 10–30, and >30 min, respectively; alkylphenols and E_3_ typically triggered early and late surges. Interestingly, a very low concentration of BPA (10^−14^ M) yielded a similar two-peak response, but a higher concentration (1 nM) induced a three-peak response like that of E_1_ and E_2_. Both BPA concentrations were typical of environmental exposures and, along with ineffective midrange doses, also illustrated the nonmonotonic dose–response relationship characteristic of many estrogenic compounds.

When physiologic estrogens and xenoestrogens were combined, the response pattern generally shifted to a single major peak at an intermediate time. Xenoestrogens that caused a strong response when administered alone at a particular point in time or concentration tended to inhibit ERK activation in response to a physiologic estrogen. But at other times or concentrations, the same xenoestrogen might cause a weak response on its own, in which case it would tend to enhance ERK phosphorylation in response to physiologic estrogens.

There were exceptions to these general patterns, however, which highlights the need to study effects of individual xenoestrogens at different points in time, at varying concentrations, and in different tissues. The effect of shifts in the patterns of ERK activation are only just beginning to be explored, although it is known that these patterns constitute an important component of information flow within a cell. The correct flow of information is likely to be especially critical during windows of vulnerability that are based in part on life stage.

